# Supplementary figures and images for: Longitudinal genome-wide association study reveals early QTL that predict biomass accumulation under cold stress in sorghum
Source: Front Plant Sci. 2024 May 14;15:1278802. doi: 10.3389/fpls.2024.1278802 (PMC11130433; doi:10.3389/fpls.2024.1278802)

**Fresh  
Weight**

$$y = 22740 + 13181x$$
$$r^2 = 0.698$$

**Biomass 51  
DAP**

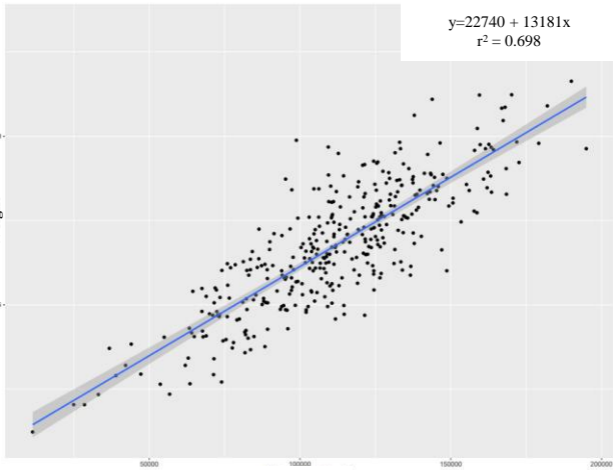

Supplement: Supplementary File S1 — Table of Bioenergy Association Panel accessions used in this study (adapted from Brenton et al., 2016) with image-derived phenotypic data. [file DataSheet_1.pdf]

# SNP 7:2,934,702

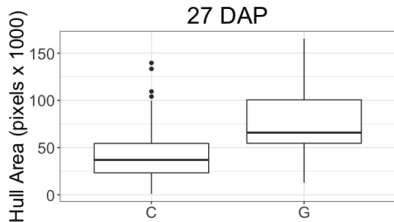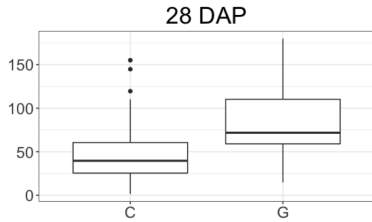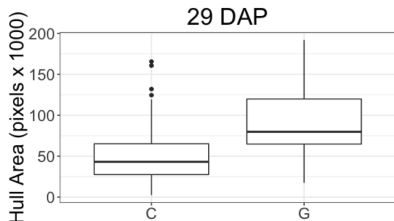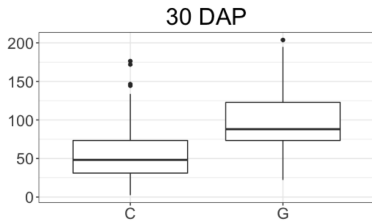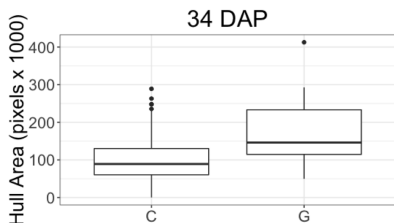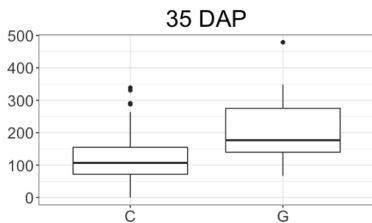

Supplement: Supplementary File S5 — Table ranking accessions for each trait at 51 DAP. [file DataSheet_5.pdf]

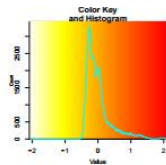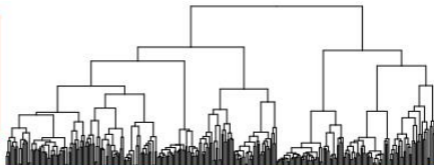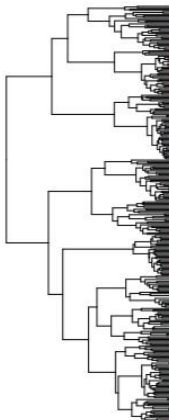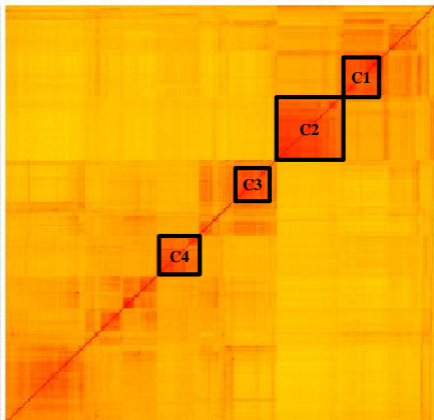

Supplement: Supplementary File S7 — Cluster analysis for biomass, height, hull area, and RGR. [file DataSheet_7.pdf]
