# Supplementary material for: Longitudinal genome-wide association study reveals early QTL that predict biomass accumulation under cold stress in sorghum
Source: Front Plant Sci. 2024 May 14;15:1278802. doi: 10.3389/fpls.2024.1278802 (PMC11130433; doi:10.3389/fpls.2024.1278802)
Supplement: Supplementary File S2 — Heatmap of a kinship matrix showing correlation analysis among the 369 BAP accessions. The color histogram shows the distribution of coefficients of coancestry values in the whole kinship matrix. The color scale shows the degree of correlation (white-yellow, low correlation; orange-red, strong correlation). C1: 25/29 accessions from Ethiopia, 26/29 photoperiod-sensitive. C2: 47/48 accessions from Ethiopia. C3: All photoperiod-insensitive accessions. C4: All cellulosic accessions; 29/30 photoperiod-sensitive. [file DataSheet_2.pdf]

a

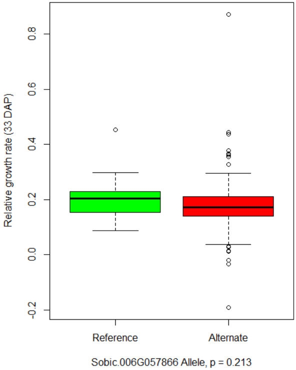

b

Chromosome	Position	Predicted Impact	Reference Allele	Alternate Allele	Predicted Effect	% Homozygous Reference[i]	% Heterozygous Lines[i]	% Homozygous Alternate[i]
Chr06	40312463	MODERATE	T	G	Missense variant	14.1	1.7	84.0
Chr06	40314154	MODIFIER	G	GTTA	Intron variant	81.8	2.8	15.2
Chr06	40314195	MODIFIER	C	G	Intron variant	80.9	1.4	17.4
Chr06	40314202	MODIFIER	A	T	Intron variant	33.7	3.3	62.7
Chr06	40314404	LOW	T	TTA	Splice region variant	68.0	4.4	26.5
Chr06	40314507	HIGH	T	C	Stop lost	3.6	0.6	94.8
Chr06	40314672	LOW	A	T	Splice region variant	90.9	0.8	8.0
Chr06	40315023	MODIFIER	T	G	3' UTR variant	18.2	2.5	79.0
Chr06	40315032	MODIFIER	A	T	3' UTR variant	90.9	0.8	8.0
Chr06	40315654	MODIFIER	G	C	3' UTR variant	18.5	2.2	79.0
Chr06	40315834	MODIFIER	A	T	3' UTR variant	80.7	1.1	18.0
Chr06	40316602	MODIFIER	A	ACGT	3' UTR variant	90.6	1.1	7.7
