# Supplementary material for: Longitudinal genome-wide association study reveals early QTL that predict biomass accumulation under cold stress in sorghum
Source: Front Plant Sci. 2024 May 14;15:1278802. doi: 10.3389/fpls.2024.1278802 (PMC11130433; doi:10.3389/fpls.2024.1278802)

a

Chromosome	Position	Predicted Impact	Reference Allele	Alternate Allele	Predicted Effect	% Homozygous Reference[i]	% Heterozygous Lines[i]	% Homozygous Alternate[i]
Chr07	2934028	MODERATE	T	C	Missense variant	29.7	2.6	67.7
Chr07	2934031	MODERATE	A	G	Missense variant	86.8	1.6	11.6
Chr07	2934040	MODERATE	A	T	Missense variant	38.5	2.9	58.6
Chr07	2934099	MODERATE	A	C	Missense variant	39.4	2.8	57.8
Chr07	2934128	MODERATE	C	A	Missense variant	79.4	0.7	19.9
Chr07	2934129	MODERATE	C	G	Missense variant	79.7	0.7	19.6
Chr07	2934187	MODERATE	C	T	Missense variant	87.8	2.1	10.1
Chr07	2934291	MODERATE	CCCG	C	Disruptive in frame deletion	42.2	3.3	54.5

b

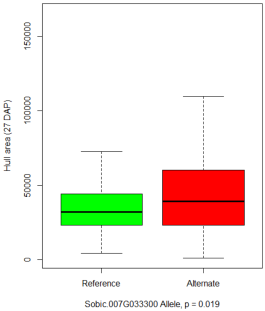

Supplement: Supplementary File S4 — Heritability of Traits. [file DataSheet_4.pdf]
