# Supplementary material for: Longitudinal genome-wide association study reveals early QTL that predict biomass accumulation under cold stress in sorghum
Source: Front Plant Sci. 2024 May 14;15:1278802. doi: 10.3389/fpls.2024.1278802 (PMC11130433; doi:10.3389/fpls.2024.1278802)
Supplement: Supplementary File S13 — Scatterplot and regression show the correlation of area at 51 DAP to endpoint fresh weight collected on 56 DAP. [file Presentation_1.pptx]

## Slide 1
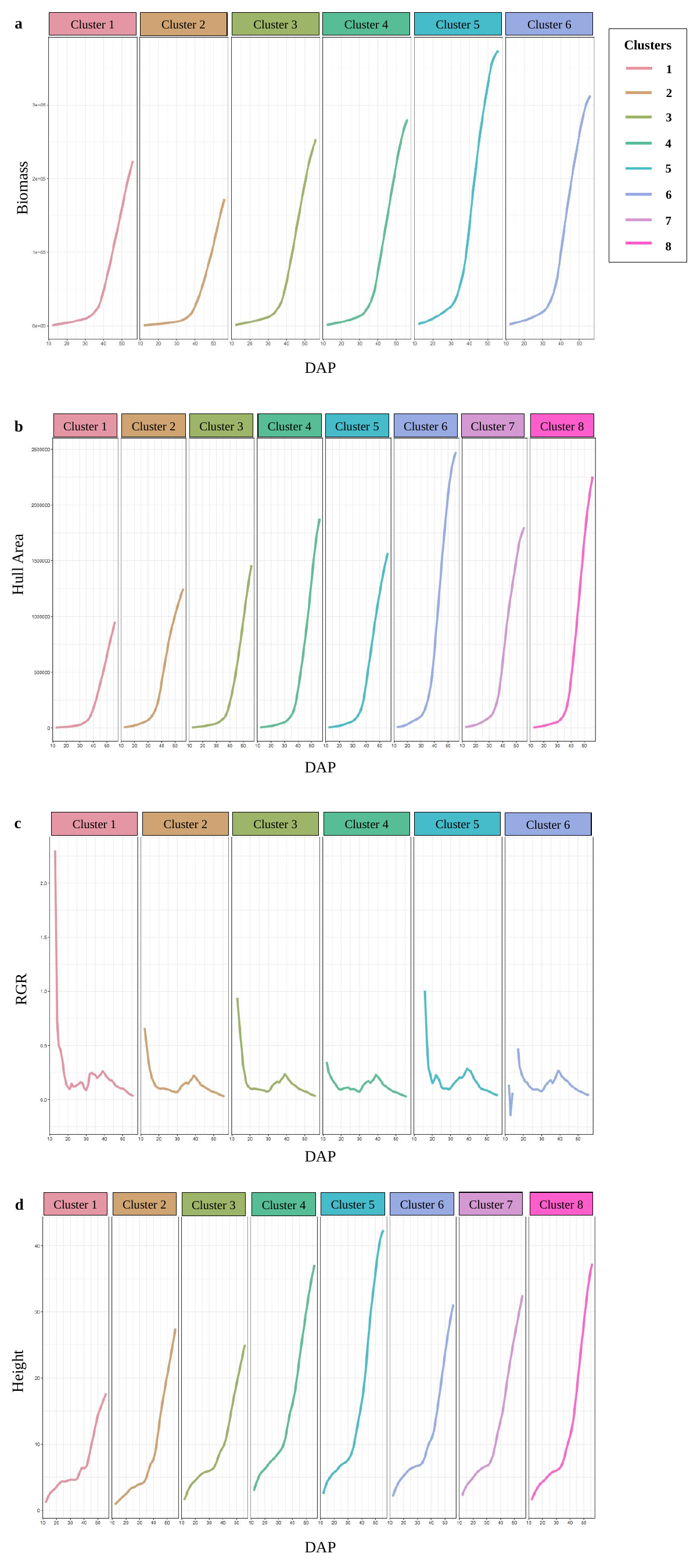

a
Cluster 6
Cluster 5
Cluster 4
Cluster 3
Cluster 1
Cluster 2
Clusters
1
2
3
4
5
Biomass
6
7
8
DAP
b
Cluster 1
Cluster 7
Cluster 8
Cluster 2
Cluster 3
Cluster 4
Cluster 5
Cluster 6
Hull Area
DAP
c
Cluster 5
Cluster 3
Cluster 4
Cluster 1
Cluster 2
Cluster 6
RGR
DAP
d
Cluster 8
Cluster 7
Cluster 1
Cluster 2
Cluster 5
Cluster 6
Cluster 3
Cluster 4
Height
DAP
